# Oral fluorouracil vs vinorelbine plus cisplatin as adjuvant chemotherapy for stage II‐IIIA non‐small cell lung cancer: Propensity score‐matched and instrumental variable analyses

**DOI:** 10.1002/cam4.1725

**Published:** 2018-08-27

**Authors:** Hirokazu Urushiyama, Taisuke Jo, Hideo Yasunaga, Nobuaki Michihata, Hiroki Matsui, Wakae Hasegawa, Hideyuki Takeshima, Yukiyo Sakamoto, Yoshihisa Hiraishi, Akihisa Mitani, Kiyohide Fushimi, Takahide Nagase, Yasuhiro Yamauchi

**Affiliations:** ^1^ Department of Respiratory Medicine Graduate School of Medicine The University of Tokyo Tokyo Japan; ^2^ Department of Health Services Research Graduate School of Medicine The University of Tokyo Tokyo Japan; ^3^ Department of Clinical Epidemiology and Health Economics School of Public Health The University of Tokyo Tokyo Japan; ^4^ Department of Health Policy and Informatics Tokyo Medical and Dental University Graduate School of Medicine Tokyo Japan

**Keywords:** adjuvant chemotherapy, non**‐**small cell lung cancer, oral fluorouracil, UFT, S‐1, vinorelbine plus cisplatin

## Abstract

**Background:**

Adjuvant chemotherapy with vinorelbine plus cisplatin (VNR/CDDP) is a standard regimen for treatment of postoperative stage II‐IIIA non**‐**small cell lung cancer (NSCLC). However, oral fluorouracil offers a feasible alternative adjuvant chemotherapeutic regimen. We compared the prognoses of patients with NSCLC treated with adjuvant chemotherapy with either VNR/CDDP or oral fluorouracil.

**Methods:**

We identified patients with stage II‐IIIA NSCLC who underwent lung surgery followed by adjuvant chemotherapy with VNR/CDDP (n = 384) or oral fluorouracil (n = 268) between July 2010 and March 2015, using the national Japanese inpatient and outpatient Diagnosis Procedure Combination database. We compared recurrence‐free survival between the groups by multivariable Cox regression analysis for one‐to‐one propensity score‐matched patients and by instrumental variable analysis.

**Results:**

Younger patients and patients with positive N2 nodes were more likely to receive VNR/CDDP, while older patients and those with T3N0 classification were more likely to receive oral fluorouracil. Among 172 pairs of propensity‐matched patients, time to adjuvant chemotherapy was shorter for oral fluorouracil compared with VNR/CDDP. Oral fluorouracil was also significantly associated with improved recurrence‐free survival compared with VNR/CDDP, according to multivariable Cox regression analysis (hazard ratio, 0.41; 95% confidence interval, 0.26‐0.64). Instrumental variable analysis showed a similar relationship (hazard ratio, 0.19; 95% confidence interval, 0.038‐0.92).

**Conclusions:**

On a large nationwide cohort, adjuvant chemotherapy with oral fluorouracil prolonged recurrence‐free survival in patients with postoperative stage II‐IIIA NSCLC, compared with VNR/CDDP. Oral fluorouracil may thus be a useful alternative to VNR/CDDP for the adjuvant treatment of these patients.

## 1 INTRODUCTION

Non**‐**small cell lung cancer (NSCLC) remains one of the most common cancers and the leading cause of cancer‐related mortality worldwide. Initial treatment for patients with early‐stage NSCLC involves complete surgical resection, while appropriate adjuvant chemotherapy has been shown to improve patient survival.^1^, ^2^, ^3^

Adjuvant vinorelbine plus cisplatin (VNR/CDDP) prolonged overall survival among patients with completely resected pathological stage II NSCLC in the JBR10 trial^1^ and in patients with stage II and stage IIIA NSCLC in the Adjuvant Navelbine International Trialist Association (ANITA) trial.^2^ Adjuvant VNR/CDDP is thus the accepted standard chemotherapy for patients with completely resected pathological stage II and stage IIIA NSCLC. However, not all patients with stage II and IIIA NSCLC are eligible for postoperative VNR/CDDP for various reasons, including poor performance status, severe renal dysfunction, or patient refusal because of adverse effects. Evidence regarding the survival benefits of adjuvant chemotherapies other than VNR/CDDP in patients with completely resected pathological stage II and IIIA NSCLC is scarce, and patients unable to receive VNR/CDDP may thus not receive postoperative chemotherapy, despite the high recurrence rate after surgery.

UFT and S‐1 are oral fluorouracil anticancer drugs. UFT improved survival in patients with completely resected pathological stage T2N0M0 (stage IB) adenocarcinoma of the lung.^3^ S‐1 was developed to reduce the gastrointestinal toxic effects of fluorouracil^4^ and has been approved for the treatment of NSCLC in Japan since 2004. The feasibility of adjuvant chemotherapy with S‐1 in patients with completely resected pathological stage IB‐IIIA NSCLC was demonstrated in two clinical trials.^5^, ^6^ However, the efficacies of adjuvant oral fluorouracil and VNR/CDDP have not been compared in patients with completely resected pathological stage II and IIIA NSCLC. Myelosuppression can be fatal and is a serious concern in some patients receiving adjuvant chemotherapy with VNR/CDDP. Rates of up to 55% for grade ≥4 neutropenia and up to 9% for febrile neutropenia have been reported in VNR/CDDP, while no incidences of grade ≥4 neutropenia have been reported in patients with stage IB‐IIIA NSCLC receiving adjuvant chemotherapy with S‐1. Reduced toxicity is thus an important requirement of adjuvant chemotherapy.

This study aimed to compare the prognoses of patients with postoperative stage II‐IIIA NSCLC treated with adjuvant chemotherapy with oral fluorouracil anticancer drugs or VNR/CDDP, by analyzing data from a national inpatient and outpatient database in Japan.

## 2 MATERIALS AND METHODS

### 2.1 Data source

We conducted a retrospective cohort study using the Diagnosis Procedure Combination (DPC) database,^7^ which is a national inpatient database covering approximately 50% of acute‐care inpatients, combined with outpatient data. The DPC database includes data on patient age, sex, body height and weight (body mass index), primary diagnosis, TNM classification, Charlson comorbidity index, Barthel index, operative procedures, chemotherapy drugs, and radiotherapy during hospitalization, discharge status, medication and treatment (including radiotherapy) in the outpatient setting, and prefecture code. This study was approved by the Institutional Review Board of The University of Tokyo. The board waived the requirement for informed patient consent because of the anonymous nature of the data.

### 2.2 Patient selection

We collected data for patients with NSCLC defined by the International Statistical Classification of Diseases and Related Health Problems, 10th revision (ICD‐10) codes C340, C341, C342, C343, C348, who underwent surgery to remove one or more lung lobes because of malignant lung cancer between July 2010 and March 2015. We further selected NSCLC patients with TNM stage II‐IIIA based on the 7th edition of the TNM classification for lung cancer.^8^ We excluded patients aged ≤17 years at the time of lung surgery, patients who received more than one surgery, and patients with a diagnosis of distant metastasis (ICD‐10 codes C40, C41, C71, C72, C77, C787, C793, and C797) before surgery. To exclude patients who received neoadjuvant therapy and adjuvant radiotherapy, we excluded patients who received radiotherapy or chemotherapy before surgery or radiotherapy within 90 days after surgery. Patients who received adjuvant chemotherapy were defined as those who started a particular adjuvant chemotherapy regimen within 90 days after surgery, without starting drugs for any different regimens. We considered the first and last days of UFT or S‐1 use, and for VNR/CDDP treatment cycles, we counted the number of simultaneous injections of the two drugs within 270 days after surgery. We also considered other drugs that may be used to treat lung cancer (paclitaxel, gemcitabine, pemetrexed, docetaxel, vinblastine, vindesine, mitomycin, amrubicin, ifosfamide, cyclophosphamide, doxorubicin, etoposide, irinotecan, nogitecan, bevacizumab, gefitinib, erlotinib, afatinib, osimertinib, crizotinib, alectinib, ceritinib, nivolumab, and pembrolizumab).

### 2.3 Primary outcome

The primary outcome of this study was recurrence‐free survival (RFS), defined as the time to any first event, including relapse or death, after lung surgery. Relapse was defined as a diagnosis of cancer relapse or distant metastasis, receipt of radiotherapy or gamma knife therapy, or switch from adjuvant chemotherapy to any chemotherapy, apart from a switch from CDDP to carboplatin in the VNR/CDDP regimen, which was not considered to indicate a relapse.

### 2.4 Statistical analysis

We performed one‐to‐one propensity score‐matching analysis between patients treated with VNR/CDDP and those treated with oral fluorouracil agents, to account for differences in baseline characteristics. We estimated the propensity scores by fitting a logistic regression model for receipt of oral fluorouracil as a function of patient demographics, including age, sex, body mass index, Charlson comorbidity index, activity of daily life scale (Barthel index), smoking index, T and N factors, comorbidities on admission for lung surgery, and geographical area of residence, as well as hospital factors such as hospital volume of lung cancer surgery (under 99, 100‐199, 200‐299, or over 300 cases of surgery during the study period, divided to give similar numbers of hospitals in each category), and teaching hospital or not. The C‐statistic for evaluating goodness of fit was calculated. A caliper width was set at 20% of the standard deviation. Matched pairs were created without replacement. Patient characteristics were compared between the two groups using the standardized difference after matching, with an absolute standardized difference >0.1 considered to indicate a significant imbalance in a covariate. The periods from surgery to the initiation of adjuvant chemotherapy were compared between the two groups using the Mann‐Whitney *U* test. RFS was compared between the VNR/CDDP and oral fluorouracil groups in the propensity‐matched patients using multivariable Cox regression analysis.

Propensity score analyses cannot remove hidden biases caused by unmeasured confounders, and we therefore confirmed the propensity score analysis results by instrumental variable analysis. Instrumental variable analysis is a pseudorandomization process that allows the data to be controlled for unmeasured confounders. Instrumental variables are assumed to be highly correlated with the treatment assignment, not correlated with any measured or unmeasured patient backgrounds, and not to affect patient outcomes, except through the study treatment.^9^ Physicians at different hospitals may have preferences for certain chemotherapies; when hospitals show strong consistency in terms of adjuvant chemotherapy for NSCLC, the choice of chemotherapy regimen is presumably independent of the individual's characteristics. According to this theory, adjuvant chemotherapy with VNR/CDDP in patients with stage I NSCLC may have depended on the hospital at which the patient was treated, rather than on their specific risk factors. Under these conditions, a hospital's preference for adjuvant VNR/CDDP in patients with stage I NSCLC was considered as an instrumental variable, even in the presence of unmeasured confounders. In the current instrumental variable analysis, we calculated the total frequency of VNR/CDDP administration in patients with postoperative stage I NSCLC for each hospital, and then divided the frequency for each hospital by the frequency in the hospital that administered VNR/CDDP most frequently, and defined it as the instrumental variable. We used the F test as a weak‐instrument identification test, and an F‐statistic <10 was considered as a weak instrumental variable.^10^ We used this instrumental variable in a two‐stage residual inclusion method to compute the hazard ratios (HRs) for cancer recurrence between the two groups in the multivariable Cox regression model, for robustness.

All statistical analyses were performed using SPSS version 22.0 (IBM SPSS Inc., Armonk, NY, USA) and Stata version 15.1 (StataCorp, College Station, TX, USA). A two‐tailed significance level of 0.05 was used in all statistical analyses.

## 3 RESULTS

### 3.1 Patient characteristics

**Figure 1:**
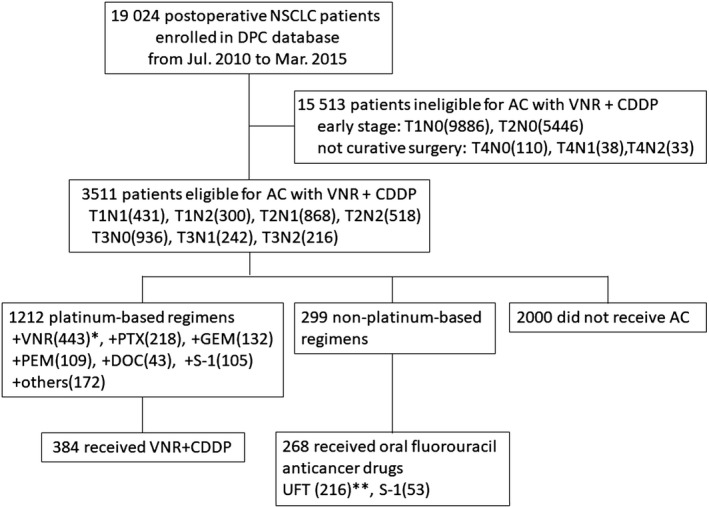
Consort diagram. *Ten of 443 patients with VNR plus platinum overlapped with PTX, GEM, PEM, DOC, or S‐1. **One patient with UFT overlapped with S‐1. AC, adjuvant chemotherapy; CDDP, cisplatin; VNR, vinorelbine; PTX, paclitaxel; GEM, gemcitabine; PEM, pemetrexed; DOC, docetaxel

We selected 19024 postoperative NSCLC patients with TNM stage T1‐4, N0‐3, and M0, based on the 7th edition of the TNM classification for lung cancer.^8^ Among 3511 stage II‐IIIA patients who underwent surgery and were potentially eligible for adjuvant chemotherapy using VNR/CDDP, we identified 384 patients who actually received VNR/CDDP and 268 patients who received an oral fluorouracil anticancer drug. A Consort flow diagram of the study is shown in Figure 1. After one‐to‐one propensity score matching, 172 pairs of VNR/CDDP and oral fluorouracil patients were selected. The C‐statistic for goodness of fit was 0.75 in the propensity score model.

The baseline characteristics of all patients (n = 652) and the propensity score‐matched patients (n = 344) are shown in Table 1. Among all patients, there were more younger patients (18‐64 years old) in the VNR/CDDP group and more older patients (≥75 years) in the oral fluorouracil group. In terms of TNM classification, there were more T3N0 patients in the oral fluorouracil group and more N2 patients in the VNR/CDDP group. There were more patients with dependent status in activities of daily life, according to the Barthel index, in the oral fluorouracil group. These imbalances in baseline patient characteristics were corrected among the propensity‐matched patients. The comorbidities on admission for lung surgery are presented in Table 2. There were more patients with chronic obstructive pulmonary disease in the VNR/CDDP group, but this imbalance was corrected after propensity score matching.

**Table 1:** Baseline characteristics of patients receiving adjuvant vinorelbine plus cisplatin or oral fluorouracil, before and after propensity score matching

Characteristic	All patients					Propensity‐matched patients				
	VNR/CDDP (N* *=* *384)		Fluorouracil (N* *=* *268)		SD	VNR/CDDP (N* *=* *172)		Fluorouracil (N* *=* *172)		SD
	N	%	N	%		N	%	N	%	
Age (y)										
18‐64	183	47.7	67	25.0	−0.490	52	30.2	57	33.1	0.071
65‐74	177	46.1	118	44.0	−0.036	97	56.4	95	55.2	−0.022
≥75	24	6.3	83	31.0	0.669	23	13.4	20	11.6	−0.067
Sex (male)	268	69.8	188	70.1	0.011	120	70.0	124	72.1	0.057
TNM classification										
Stage II										
T3N0	87	22.7	84	31.3	0.193	47	27.3	51	30.0	0.059
T1N1	47	12.2	39	14.6	0.074	26	15.1	24	14.0	−0.045
T2N1	82	21.4	67	25.0	0.090	43	25.0	40	23.3	−0.048
Stage IIIA										
T3N1	19	4.9	13	4.9	−0.006	8	4.7	7	4.1	−0.026
T1N2	54	14.1	17	6.3	−0.259	13	7.6	11	6.4	−0.042
T2N2	65	16.9	35	13.1	−0.111	26	15.1	28	16.3	0.037
T3N2	30	7.8	13	4.9	−0.123	9	5.2	11	6.4	0.053
Body mass index (kg/m^2^)										
<18.5	22	5.7	14	5.2	−0.023	6	3.5	9	5.2	0.088
18.5‐24.9	256	66.7	175	65.3	−0.036	116	67.4	112	65.1	−0.058
≥25	104	27.1	79	29.5	0.050	50	29.1	51	29.7	0.020
Missing	2	0.5	0	0.0	NC	0	0.0	0	0.0	NC
Charlson comorbidity index										
0‐2	252	65.6	188	70.1	0.095	111	64.5	111	64.5	0.004
≥3	132	34.4	80	29.9	−0.095	61	35.5	61	35.5	−0.004
Activity of daily life (Barthel index)										
Independent (100)	375	97.7	255	95.1	−0.134	169	98.3	168	97.7	−0.047
Dependent (≤95)	4	1.0	10	3.7	0.176	3	1.7	2	1.2	0.047
Missing	5	1.3	3	1.1	−0.017	0	0.0	2	1.2	NC
Smoking index										
Never	107	27.9	75	28.0	−0.001	51	30.0	46	26.7	−0.058
<10 pack‐years	12	3.1	12	4.5	0.078	5	2.9	4	2.3	−0.035
≥10 pack‐years	262	68.2	170	63.4	−0.098	113	65.7	119	69.2	0.067
Missing	3	0.8	11	4.1	0.216	3	1.7	3	1.7	0.002

SD, standardized difference; VNR/CDDP, vinorelbine plus cisplatin; NC, not calculable.

**Table 2:** Comorbidities on admission for lung surgery in stage II‐IIIA non‐small cell lung carcinoma patients

Comorbidity	All patients					Propensity‐matched patients				
	VNR/CDDP (N* *=* *384)		Fluorouracil (N* *=* *268)		SD	VNR/CDDP (N* *=* *172)		Fluorouracil (N* *=* *172)		SD
	N	%	N	%		N	%	N	%	
Chronic obstructive pulmonary disease	104	27.1	52	19.4	−0.180	44	25.6	42	24.4	−0.020
Interstitial pneumonia	12	3.1	4	1.5	−0.095	2	1.2	2	1.2	0.001
Congestive heart failure	18	4.7	17	6.3	0.071	12	7.0	9	5.2	−0.097
Ischemic heart disease	28	7.3	21	7.8	0.019	12	7.0	15	8.7	0.068
Tachycardia	66	17.2	48	17.9	0.017	28	16.3	30	17.4	0.036
Chronic liver disease	13	3.4	6	2.2	−0.070	4	2.3	6	3.5	0.071
Chronic renal failure	6	1.6	4	1.5	−0.006	1	0.6	1	0.6	0.001
Past history of other cancer	12	3.1	13	4.9	0.087	7	4.1	5	2.9	−0.061

SD, standardized difference; VNR/CDDP, vinorelbine plus cisplatin.

### 3.2 Chemotherapy compliance

The details of adjuvant chemotherapy are shown in Table 3. The mean number of cycles of adjuvant VNR/CDDP group was 3.0 (standard deviation 1.4), and the median duration of adjuvant oral fluorouracil was 127 days (interquartile range 29‐292). These were similar in all patients and in the propensity‐matched patients. The time to adjuvant chemotherapy was significantly shorter in the oral fluorouracil compared with the VNR/CDDP group, among both unmatched and propensity‐matched patients.

**Table 3:** Adjuvant chemotherapy with vinorelbine plus cisplatin or oral fluorouracil

Treatment period	All patients				Propensity‐matched patients			
	VNR/CDDP (N* *=* *384)		Fluorouracil (N* *=* *268)		VNR/CDDP (N* *=* *172)		Fluorouracil (N* *=* *172)	
	Mean	SD	Median	IQR	Mean	SD	Median	IQR
Cycles	3.0	1.4			3.0	1.4		
Days			127	29−292			152	43−297

Time from surgery to starting AC	Median	IQR	Median	IQR	*P*	Median	IQR	Median	IQR	*P*
Days	51	43−64	44	32−55	<0.001	51	43−65	45	35−58	<0.001

SD, standard deviations; IQR, interquartile range; AC, adjuvant chemotherapy; VNR/CDDP, vinorelbine plus cisplatin.

### 3.3 RFS

Among the propensity‐matched patients, adjuvant oral fluorouracil was significantly associated with improved RFS compared with VNR/CDDP, according to multivariable Cox regression analysis (HR, 0.41; 95% CI, 0.26‐0.64) after adjustment for stage II and stage IIIA. There was no difference in RFS between patients with stage II and stage IIIA NSCLC (HR, 1.08; 95% CI, 0.69‐1.68) after adjusting for adjuvant chemotherapy. The F‐statistic in the instrumental variable analysis was 24.5, and RFS was significantly longer in the oral fluorouracil compared with the VNR/CDDP group (HR, 0.19; 95% CI, 0.038‐0.92).

## 4 DISCUSSION

The current study showed that adjuvant chemotherapy with an oral fluorouracil anticancer drug was significantly associated with better RFS in patients with postoperative stage II‐IIIA NSCLC, compared with adjuvant VNR/CDDP. To the best of our knowledge, this study provides the first evidence regarding treatment outcomes in patients with stage II‐IIIA NSCLC requiring adjuvant chemotherapy in a nationwide clinical setting.

A previous study that analyzed data from the Surveillance, Epidemiology, and End Results Program of the National Cancer Institute reported that postoperative radiotherapy was associated with improved survival of patients with stage II and III NSCLC with N2 nodal disease, but not N0 or N1 nodal disease, who underwent lung surgery.^11^ Another study using the same database reported that platinum‐based adjuvant chemotherapy was associated with reduced mortality in elderly stage I NSCLC patients with tumors ≥4 cm compared with resection alone.^12^ The current study had the advantage of using a database that included information not available in these earlier studies, that is, information on particular chemotherapy drugs, body mass index, and Barthel index. We were therefore able to compare the prognostic effects of adjuvant chemotherapy with oral fluorouracil and VNR/CDDP, after controlling for patient characteristics by propensity score‐matching and instrumental variable analysis.

We conducted a quasiexperimental study using two different pseudorandomization techniques (propensity score‐matching and instrumental variable analysis) to compare the efficacies of different adjuvant chemotherapies. Instrumental variable analysis allowed us to control for hidden biases caused by unmeasured confounders, which were not removed by propensity score‐matching and multivariate regression analysis. Instrumental variable analysis also showed a preferable effect of oral fluorouracil with a lower point estimate of risk compared with propensity score analysis. Instrumental variable analysis only evaluates the effectiveness of the treatment in the group changing treatment according to the instrumental variable (ie, local average treatment effect).^13^ The effect size may thus be this large in a population of patients who might have received oral fluorouracil if they had not visited a hospital that tended to use CDDP/VNR after radical surgery. The demonstrated advantage of oral fluorouracil compared with VNR/CDDP in the current study may thus provide robust evidence for its use as postoperative adjuvant chemotherapy in patients with stage II‐IIIA NSCLC.

According to the present study, the time to adjuvant chemotherapy was significantly shorter in patients receiving oral fluorouracil compared with VNR/CDDP. A previous meta‐analysis reported that a delay in administering adjuvant chemotherapy was associated with poorer survival among patients with resected colorectal cancer 14 and breast cancer.^15^ We therefore speculated that the favorable RFS among the oral fluorouracil group may have been partly associated with the shorter time to adjuvant chemotherapy after surgery. We also found that patients in the oral fluorouracil group received adjuvant chemotherapy for a longer period, which could also help to explain the more favorable RFS. One possible explanation for the shorter time to adjuvant chemotherapy and longer duration in patients receiving oral fluorouracil is its reduced toxicity compared with VNR/CDDP. Earlier studies showed that the incidences of grade ≥4 neutropenia were 55% and 0% with VNR/CDDP^16^ and S‐1,^5^, ^6^ respectively. Indeed, 72% of patients with postoperative stage IB‐IIIA NSCLC completed 48 weeks of planned adjuvant chemotherapy with S‐1,^5^ whereas only 50% of patients completed the planned four cycles of CDDP/VNR.^2^

UFT and S‐1 contain the anticancer agent tegafur, which is a prodrug of 5‐fluorouracil with additional compounds to support the effect of fluorouracil. Although UFT and S‐1 are not identical, an earlier study reported that they showed very similar efficacies and toxicities when administered as adjuvant chemotherapy for postoperative NSCLC.^17^ We therefore considered either of these drugs as oral fluorouracil.

This study had some limitations. First, we did not take account of the detailed surgical results or the severity of postoperative symptoms. Second, there may have been selection bias in the treatment arms. Furthermore, optimal surgical margins and appropriate lymph node dissection have been reported for lung cancer surgery in Japan^18^; however, the study results could have been biased if more patients in the VNR/CDDP group had positive surgical margins or incomplete lymph node resection. Third, this study was unable to use evaluations based on the central review of the diagnosis and staging of NSCLC. However, an earlier study using the DPC database showed that each 1‐ to 5‐year survival of postoperative stage IA‐IIIA NSCLC^19^ was similar to that for each corresponding pathological stage in the national lung cancer registry study of the Japanese Joint Committee for Lung Cancer Registration.^20^ Fourth, we could not identify all deaths outside the participating hospitals. Patients with end‐stage NSCLC are often discharged to home care, hospices, nursing homes, or community hospitals, but deaths after discharge are not recorded in the DPC database. However, patients who underwent surgery were likely to visit and be treated at the same hospital if they developed disease recurrence. We therefore believe that we were aware of most events related to postoperative lung cancer recurrence. Discharge to another hospital or home care was censored in the survival analyses of RFS.

## 5 CONCLUSION

The present study showed that adjuvant chemotherapy with oral fluorouracil was significantly associated with improved postoperative RFS in patients with stage II‐IIIA NSCLC, compared with VNR/CDDP, based on propensity score‐matching and instrumental variable pseudorandomization techniques. Our study also showed that the time to adjuvant chemotherapy was significantly shorter in patients receiving oral fluorouracil compared with VNR/CDDP. This was a retrospective study, and further prospective studies are therefore needed to clarify the optimal adjuvant chemotherapy in patients with postoperative NSCLC.

## CONFLICT OF INTEREST

The authors have declared no conflict of interests.
